# Recombinant antibodies against Iranian cobra venom as a new emerging therapy by phage display technology

**DOI:** 10.1590/1678-9199-JVATITD-2019-0099

**Published:** 2020-06-19

**Authors:** Ali Nazari, Maedeh Samianifard, Hadi Rabie, Abbas Zare Mirakabadi

**Affiliations:** 1 Department of Biochemistry and Proteomics, Razi Vaccine and Serum Research Institute, Agricultural Research, Education and Extension Organization (AREEO), Karaj, Iran.; 2 Department of Venomous Animals, Razi Vaccine and Serum Research Institute, Agricultural Research, Education and Extension Organization (AREEO), Karaj, Iran.

**Keywords:** Antibody phage display, Venom, Antivenom, Purification

## Abstract

**Background:**

The production of antivenom from immunized animals is an established treatment for snakebites; however, antibody phage display technology may have the capacity to delivery results more quickly and with a better match to local need. *Naja oxiana*, the Iranian cobra, is a medically important species, responsible for a significant number of deaths annually. This study was designed as proof of principle to determine whether recombinant antibodies with the capacity to neutralize cobra venom could be isolated by phage display.

**Methods:**

Toxic fractions from cobra venom were prepared by chromatography and used as targets in phage display to isolate recombinant antibodies from a human scFv library. Candidate antibodies were expressed in *E. coli* HB2151 and purified by IMAC chromatography. The selected clones were analyzed in *in vivo* and *in vitro* experiments.

**Results:**

Venom toxicity was contained in two fractions. Around a hundred phage clones were isolated against each fraction, those showing the best promise were G12F3 and G1F4. While all chosen clones showed low but detectable neutralizing effect against *Naja oxiana* venom, clone G12F3 could inhibit PLA_2_ activity.

**Conclusion:**

Therefore, phage display is believed to have a good potential as an approach to the development of snake antivenom.

## Background

Animal bites are a major source of global morbidity and mortality. The health impact of snake envenomation depends on the species, the circumstances under which a person is bitten, and access to appropriate health care. According to data from the World Health Organization, around five million people are bitten by snakes annually, a significant number in Africa and Asia. Nearly half of all snake bites give rise to envenomation and around 100,000 deaths occur every year and a far greater number of people suffer severe and long-lasting health impacts [1]. 

Snake venoms comprise a complex mixture of different proteins, enzymes, and bioactive peptides and vary widely in their composition and mode of action between different snake species [2, 3]. The biochemical and pharmacological properties of snake venom have been extensively studied [4, 5]. Antivenoms are traditionally used to treat victims of envenomation, the first report of the use of hyperimmunized serum by Chalmette dates back over 120 years [6]. The production of these neutralizing agents in animals such as horses and sheep continues, and progress in downstream processing has achieved decreases in the side effects of these therapeutics. 

Recently, the potency of antibodies from camels has been explored [7] and recombinant antibody technology has begun to gain ground [8]. Antivenoms can be effective as polyclonal antisera manufactured and purified in bulk [9], but monospecific or polyspecific fragments such as F(ab’)2 or Fab have advantages through the reduction in non-specific binding, the failure to interact with cellular Fc receptors, and improved tissue penetration because of their small size [10]. 

Phage display technology is an established system introduced by G. Smith [11] for the selection of molecules that are able to bind specifically to a target. The technology is based upon simple principles: the cloning of coding sequences into the DNA of bacteriophage; the presentation of encoded sequences such as an antibody at the surface of the filamentous phage; the capture of phage from libraries of candidate binders on surfaces coated with a target of interest. The association of genotype (sequences carried within the virus) and phenotype (the binding molecule displayed at the viral surface, most commonly as a fusion with pIII, a coat protein of M13 phage) makes the system ideal for the extraction of antibodies from large natural or synthetic libraries by *in vitro* panning against the desired target and rounds of selection and amplification in the *E. coli* host [12, 13]. 

Phage display technology is beginning to be used for selecting antibodies *versus* the toxins of snake venoms [14]. The first report described selected recombinant antibodies against crotoxin from an antibody library produced in mice [15]. Recombinant scFvs antibodies from a non-immune human library were found to neutralize phospholipase A_2_ in crotoxin, suggesting that these molecules have immunotherapeutic potential [16]. Following these early reports, recombinant antibodies have been selected against diverse toxins from venomous animals such as scorpions, spiders [17] and insects [18] and the system is ideal for the isolation of small antibody fragments such as scFv, or Fab or possibly F (ab’)2 with the advantages highlighted above, using immunized or naïve libraries [19] derived from humans, camels, or even chickens [20, 21, 22]. The speed and flexibility of this technology make it suited to meeting regional need. In this study, we aimed to isolate recombinant scFvs fragments from a library of randomized human antibody sequences, seeking scFvs with the capacity to neutralize the toxic fractions of the Iranian cobra *(Naja oxiana*) snake venom, a medically important species in this region of the Middle East.

## Methods

### Venom fractionation

Sephadex G-50 was used to fractionate *Naja oxiana* venom that was obtained from the Department of Venomous Animals at the Razi Vaccine and Serum Research Institute, Karaj, Iran. A column of 2 x 90 cm was equilibrated with PBS pH 7.5, loaded with 180 mg venom, and 5-mL fractions were collected at a flow rate of 50 mL/h. Protein composition was detected by absorbance measured at 280 nm to derive a chromatogram. The toxicity of all fractions was determined as detailed below. 

### Phage library and antibody selection

The Tomlinson scFv libraries contain human antibody sequences with randomized residues at critical points in the complementarity-determining regions (CDRs) of the heavy and light chains. The scFv sequences were constructed as fusions to the gene for the minor phage coat protein pIII in pIT2, a phagemid bearing an ampicillin resistance marker. Libraries were obtained from the MRC HGMP Resource Centre, UK. Libraries were maintained in *E. coli* TG1 cells, and rescued by infection with KM13 helper phage according to the manual provided with the libraries. 

The resource contained two libraries, I and J, which differed in the approach used to generate diversity in the CDRs. Library J has the greater capacity for diversity but greater chance that stop codons will occur in the reading frame for the scFv-pIII fusion. Although both libraries were used in this study, results focus on outcomes from experiments with library I. To select specific binders, the wells of a microplate (Nunc, Denmark) were coated independently with 10 µg of venom fractions three (F3) and four (F4) in PBS since these fractions were found to contain phospholipase A_2_ (PLA_2_) activity (see “Results” section). 

The micro plate was stored overnight at 4°C, then washed three times with phosphate buffer. To block residual binding capacity in each well, 2% skim milk in PBS was added and incubated at room temperature for 4 h. To each well, 10^13^ phage from the libraries was added to allow attachment of those with the capacity to bind to venom components. Plates were agitated on a shaker at room temperature for 4 h. Following this, unbound viruses were discarded by washing with phosphate buffer containing 0.1% Tween 20. The KM13 helper phage encodes a pIII mutant that is protease sensitive. Hence, to release any phage attached to venom components, 0.5 mL trypsin solution (1 mg/mL) was added. A volume of 0.25 mL of the elute from each well was incubated with *E. coli* TG1 that had been grown to an OD at 600 nm of 0.4 at 37°C. Phage infection and conversion to ampicillin resistance thereby provided an estimate of phage recovery; to retrieve this data, serial dilutions of infected bacteria were plated to TYE agar containing 100 µg/mL ampicillin and 1% glucose. Bacteria infected with the remaining phage eluate were superinfected with 10^10^ PFU of KM13 to amplify that phage recovered from the selection step. 

Newly-formed phage particles were precipitated from the supernatants of overnight cultures by addition of 20% polyethylene glycol 6000, 2.5 M NaCl, and collected by centrifugation at 3300 × *g* for 15 min. Samples of virus in the recovered precipitates were infected with *E. coli* TG1 and titration on TYE with ampicillin and glucose was used to calculate phage input to the next round of panning. In all, three rounds of selection were carried out with stocks of phage recovered at each stage stored at -70°C in 15% glycerol solution.

### Reactivity of monoclonal phage with venom fractions

Single colonies from the third round of selection against venom fractions were picked from titration plates and grown individually in 96-well flat bottom plates inoculated with 2xTY ampicillin medium. Aliquots containing 10^9^ PFUs of KM13 helper phage were added to 100 µL of each bacterial culture and grown on overnight at 37°C to generate monoclonal stocks of phage particles. Plates were centrifuged to sediment bacteria and supernatants from each well were transferred to ELISA plates pre-coated with the venom fraction used for selection (10 µg/well). Phage binding in each well was detected with 100 µL of a 1/5000 dilution of a monoclonal HRP-conjugated anti-M13 antibody and after washing, 100 µL TMB substrate. Reactions were quenched with 50 µL of 1M sulfuric acid per well and optical densities were recorded at 450 nm.

### Preparation of soluble monoclonal scFv antibodies

Soluble scFv proteins were prepared from those phages that showed high absorbance in monoclonal ELISA analysis. Viral samples from these clones were infected into the non-suppressor *E. coli* strain HB2151. In this background, translation is terminated at a stop codon at the junction of reading frames for scFv and pIII; in the TG1 suppressor strain described earlier, read-through generates a scFv-pIII fusion that integrates into the viral coat for display and selection. Cultures of infected bacteria were plated to TYE agar containing 100 µg/mL of ampicillin and 1% glucose. 

Colonies were transferred to liquid culture in flat bottomed plates, grown to an OD at 600 nm of 0.9 and protein expression was induced by addition of IPTG to 1 mM. Cultures were grown overnight at 30°C. To test for the binding of scFv to venom components, culture plates were centrifuged and supernatants from each well were transferred to pre-coated ELISA plates. Protein binding was detected with an anti c-myc-HRP conjugate (Gene script, A00863) and TMB substrate. When scaled up, culture supernatants were prepared and soluble scFv was precipitated with 60% saturated ammonium sulfate. Following dialysis, antibodies were purified by immobilized nickel affinity chromatography (IMAC) using 20 mM phosphate buffer pH 7.4, as binding buffer, and 50 mM imidazole in binding buffer for elution. Columns were regenerated with 0.2 M NiSO_4_, 50 mM EDTA. The purity of isolated antibodies was assessed by electrophoresis and their reactivity with the target (venom fractions F3 or F4) was confirmed by dot blotting. This was done by spotting venom fractions F3 or F4 to nitrocellulose membrane, applying the soluble scFv under evaluation, and detecting the binding of recombinant antibody with reagents to the c-myc tag encoded at the carboxy-termini of the scFvs. 

### SDS-PAGE analysis

Samples were analyzed to assess their protein composition and purity using sodium dodecyl sulfate-polyacrylamide gel electrophoresis [23]. Ten percent polyacrylamide gels were used for scFv analysis and 15% for chromatographic fractions from venom. A mini-PROTEAN system (Bio-Rad) was used. Separated proteins were revealed by staining gels with bromophenol blue. Protein quantification was determined by a quick start Bradford protein assay [24].

### Determination of phospholipase A_2_ activity

Phospholipase activity was estimated using a secretory phospholipase A_2_ assay kit (Abcam, Germany). Fractions separated by chromatography on Sephadex were tested for activity. The assay is based upon the production free thiols by hydrolysis of a 1, 2-dithio analogue of diheptanoyl phosphatidylcholine and detection with 5, 5’-dithio-bis-(2-nitrobenzoic acid). Assays were conducted in microtiter plates with serial dilutions of test samples and optical densities were recorded at 414 nm at minute intervals over five minutes. Enzyme activity was calculated according to the manual. 

### Toxicity and neutralization assays

To assess the toxicity of the original venom and its fractions, samples were injected intravenously to mice (NIH strain, 18-20 g, Razi Institute) in groups of five. Mortality was recorded 48 h after injection and the Sperman and Karber method applied to assess lethality values [25]. To assess the neutralizing capacity of scFvs, mixtures of phage or soluble scFv protein were prepared with crude venom from *Naja oxiana* in varying ratios by volume. After incubation for 1 h at temperature of 37°C, 0.5-mL samples were injected to mice in groups of ten and mortality followed thereafter. PBS was used as a negative (non-neutralizing) control; polyvalent antivenom prepared at the Razi Institute was used as the positive (neutralizing) control. 

### Restriction analysis of selected clones

The pIT2 vector used in library construction contained four restriction sites used for analysis for the presence and length of the heavy and light chain coding sequences in each clone. Digestion of DNA with *Nco*I and *Not* I excised the full scFv coding sequence for analysis. Digestion with *Nco*I and *Xho*I released the heavy chain component whereas SalI and *Not*I excised the light chain from the vector. Phagemid DNA was prepared from 5 mL of individual clones in *E. coli* HB2151 (QIA miniprep kit) and after digestion with restriction enzymes, products were separated on 1% agarose gels in Tris-Acetate-EDTA buffer at 100 V for around 1 h. DNA was stained with SYBR Safe (Invitrogen, S33102) and images captured under UV illumination. 

## Results

### **Fractionation of *Naja oxiana* venom**

**Figure 1.**
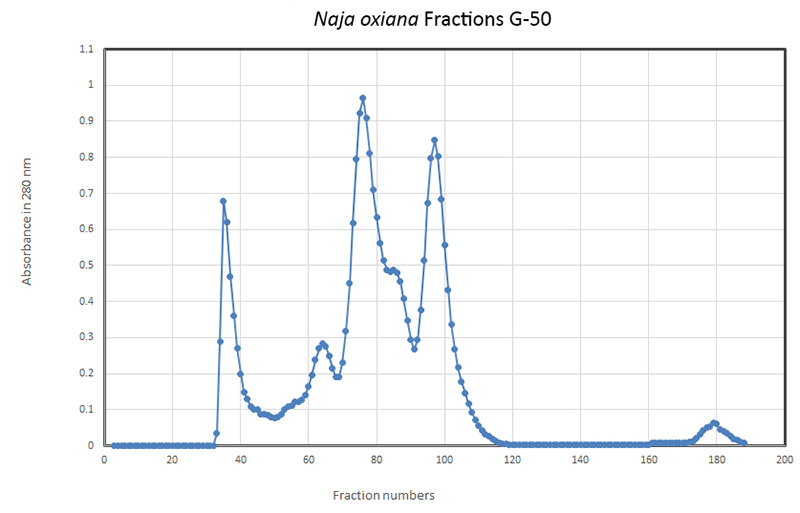
Gel filtration chromatography of *Naja oxiana* venom on Sephadex G-50. An amount of 180 mg of crude venom was separated on a 2 x 90 cm column previously equilibrated with phosphate buffer pH 7.5. Five milliliter samples were collected at a flow rate of 50 mL/h and their absorbance was measured at 280 nm. Four well-separated fractions of interest (F1, F2, F3 and F4) were taken forward for analysis.

**Figure 2.**
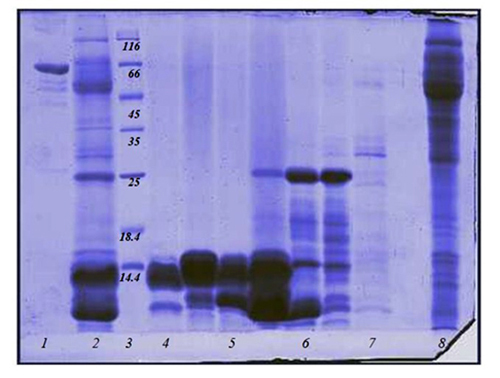
SDS-PAGE analysis of crude *Naja oxiana* venom and chromatographic fractions. Lane 1: albumin; lane 2: crude venom; lane 3: protein markers; lane 4: fraction F4; lane 5: fraction F3; lane 6: fraction F2; lane 7: fraction F5; lane 8: fraction F1.

Column chromatography on Sephadex G-50 generated four well-separated fractions from *Naja oxiana* venom, designated F1 to F4 (Figure 1). A fifth peak (designated fraction F5) eluting late from the column around sample 180 (Figure 1) was also collected. All fractions were analyzed on SDS-PAGE under non-reducing conditions. Crude venom (Figure 2, lane 2) contained proteins at a range of molecular weights, but was dominated by two proteins of less than 14 kDa. Of the fractions recovered from chromatography, Fraction 1 (lane 8) contained venom proteins at the higher end of the range, whereas Fraction 2 (lane 6) was dominated by a protein of 25 kDa (Figure 2). 

The prominent proteins of low molecular weight identified in crude venom were recovered in fractions F3 (lane 5) and F4 (lane 4). Fraction F5 did not contain any proteins of note (lane 7). Although F3 and F4 were well-separated by chromatography (Figure 1), their protein compositions did not appear to differ significantly. All fractions were assessed for PLA_2_ activity; only F3 and F4 possessed enzyme activity in this assay. The LD_50_ value for crude venom of *Naja oxiana* was calculated to be 8.7 µg/ mouse. For fractions F3 and F4, LD_50_ values of 64 and 1.7 µg/mouse were determined. On the basis of these findings, F3 and F4 were used in biopanning and antibody selection.

### Selection of scFvs against toxic venom components

After recovery of the libraries from frozen stocks, titers of phage were determined to be approximately 10^14^/mL. At each stage of selection, around 10^13^ phages were used as input. Independent selections were undertaken with libraries I and J, seeking phage with the capacity to interact with proteins contained in fractions F3 and F4. Table 1 shows the progress of selection of phage from Tomlinson library I, with the numbers of phage input to each round and output recoveries. 

**Figure 3.**
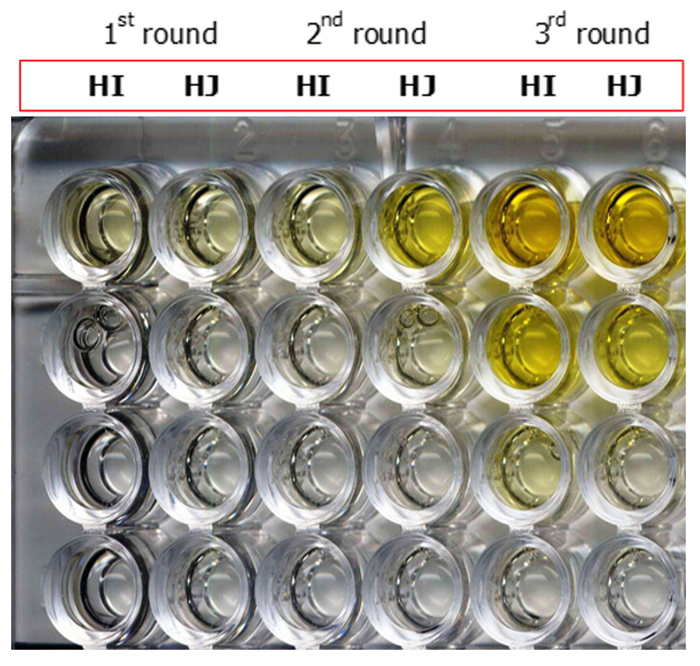
Polyclonal phage ELISA of phage selected in successive rounds of panning against fraction F3. Results from selections from library I (HI) and J (HJ) are shown. The rounds of selection are as designated on the top of the image. Overnight coating of F3 (30 µg/mL). Around 10^13^ phages into the first well of each column, then 10-fold dilution down the plate. Adding anti-M13/HRP to detect binding of virus to the target. TMB was added as substrate for HRP. Reactions were stopped with sulfuric acid (yellow).

Yields improved by approximately two orders of magnitude from round one to round three suggesting the progressive enrichment of phage with the capacity to bind to proteins in fractions F3 and F4. This was tested in polyclonal phage ELISA. In Figure 3, ELISA wells were coated with proteins contained in fraction F3, and the binding of phage recovered from three rounds of selection with libraries I and J was assessed. Initially (after the first round of selection) low signal strength indicated small numbers of virus from library I (HI) and library J (HJ) that were able to bind to target proteins. By round 3 (right side of Figure 3), ELISA signals from libraries I and J remained detectable through several 10-fold dilutions indicating specific interaction with venom proteins. Analysis progressed to monoclonal phage ELISA.

**Table 1.** Phage recovery through selection against fractions F3 and F4

Library	Library I titration (cfu/mL)		
Selection	Input	Output	Recovery
Round 1 F3	6 x 10^13^	3.6 x 10^4^	6 x 10^-8^
Round 1 F4	6 x 10^13^	1.4 x 10^5^	2 x 10^-8^
Round 2 F3	2 x 10^13^	2.7 x 10^7^	1.35 x 10^-6^
Round 2 F4	10^13^	4.4 x 10^6^	4.4 x 10^-7^
Round 3 F3	10^13^	8 x 10^7^	8 x 10^-6^
Round 3 F4	10^13^	7.1 x 10^7^	7.1 x 10^-6^

### Screening of monoclonal phages

**Figure 4.**
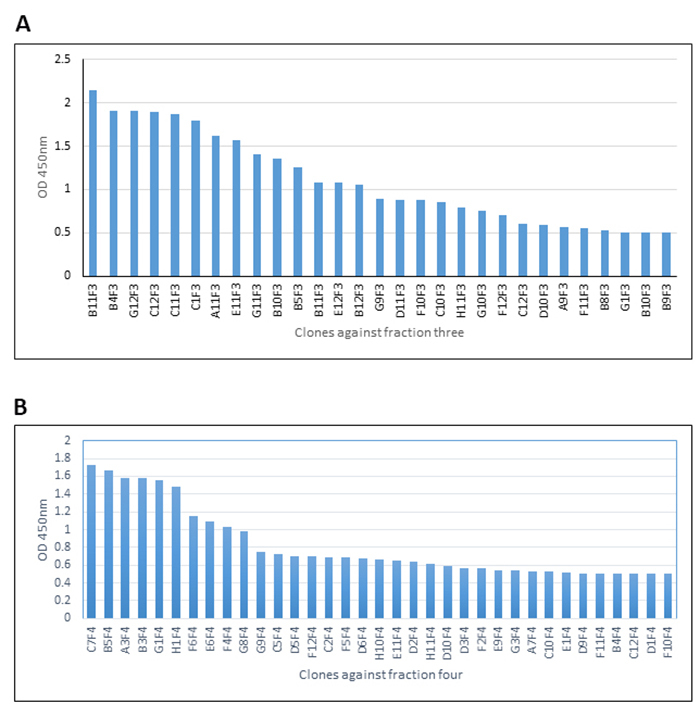
Monoclonal phage ELISA of phage isolated from antibody library I against **(A)** fraction F3 and **(B)** fraction F4.

Individual colonies from the third round of selection with libraries I and J against F3 and F4 were picked at random and monoclonal phage stocks prepared in microplates for analysis. A range of signal strength was observed in monoclonal phage ELISA against venom fraction F3 (Figure 4A) and F4 (Figure 4B). On the basis of these data, seven clones against fraction F3 and five clones against F4 were progressed for the preparation of soluble scFv antibody for further evaluation and testing in neutralization assays.

### Restriction analysis of scFv coding sequences

 In page display, the cyclical process of selection and enrichment can favor isolation of mutants from which heavy or light chain components of the scFv have been deleted. Restriction analysis of the phagemids encoding the 12 scFvs chosen for further analysis confirmed that all antivenom clones were intact: digestion of phagemid DNA with *Nco*I and *Not*I released a fragment in excess of 700 base pairs corresponding to the predicted size of the full length scFv reading frame. Fragments in excess of 300 base pairs were released by digestion with *Nco*I and *Xho*I (scFv heavy chain) and *Sal*I / *Not*I (scFv light chain; data not shown). 

### Dot blot analysis

**Figure 5.**
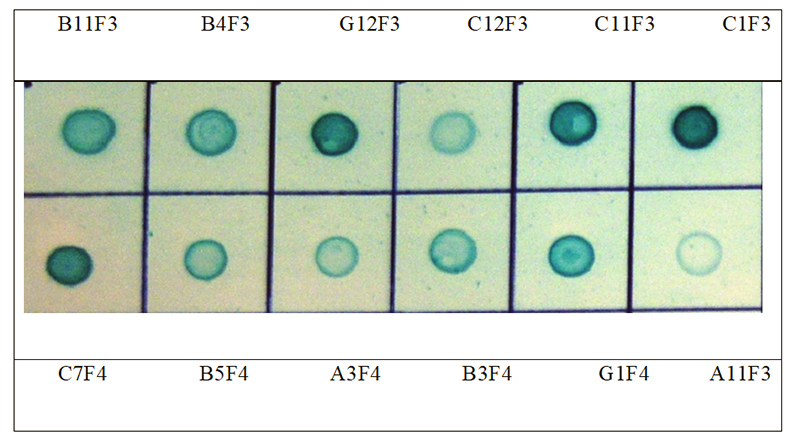
Immunoblot assay of binding of purified scFvs against their targets. Clones as designated in Figure 4.

In an alternative assay format, venom fractions F3 and F4 were spotted to nitrocellulose membrane and soluble scFv protein purified by IMAC was applied. Detection with anti-c-myc reagents revealed binding of scFvs to components of each venom fraction (Figure 5). The rank order of signal strength was in some cases at variance to that shown in Figure 4, perhaps reflecting differences of target conformation or epitope availability on nitrocellulose (Figure 5) *versus* plastic (Figure 4), or relative differences in scFv yield in microplates (Figure 4) *versus* scaled-up culture (Figure 5). For example, ELISA indicated that scFv clone B11F3 reacted very strongly with proteins in venom fraction F3, exceeding the recognition of clones G12F3, C11F3 and C1F3 (Figure 4). In contrast, dot blotting showed the signal strength from G12F3, C11F3 and C1F3 exceeded that of B11F3 (Figure 5). Similar effects were seen with scFvs against proteins in fraction F4: although C7F4 was dominant in both assays, G1F4 rose from fifth in signal strength in ELISA (Figure 4) to the second strongest of the five clones chosen for further evaluation when tested in dot blots (Figure 5). Nevertheless, all 12 scFvs retained their capacity to bind to their cognate target in dot blot analysis,

### Venom neutralization with soluble scFv proteins

As aforementioned, *Naja oxiana* crude venom and fractions F3 and F4 demonstrated significant lethal potency *in vivo*. To test whether scFvs isolated by phage display possessed neutralizing activity as well as the capacity to recognize venom components *in vitro*, monoclonal phage stocks and purified scFv antibodies were incubated at varying ratios with crude venom and tested in lethality assays. Both phage and their soluble scFv products demonstrated detectable neutralizing activity when incubated with venom prior to injection into mice. 

In preliminary experiments with purified antibody from clones C1F3 and G12F3, venom at doses of 0.5 LD_50_ was mixed with samples of each soluble scFv and injected into groups of mice. Twenty-four hours post-injection, survival was appreciably greater than in negative control groups. In contrast, neutralization was undetectable when scFv B4F3 was mixed with venom prior to injection. Further *in vitro* evaluation demonstrated that scFvs C1F3 and G12F3 were able to neutralize the PLA_2_ activity present in venom fraction F3. These *in vitro* and *in vivo* properties were also observed with scFv G1F4 directed against components in fraction F4. 

## Discussion

Significant mortality arises in Asia every year from snakebites, the estimates renge between about 30,000 and 50,000 deaths [26]. Preparing effective and cheap antivenom therapies has been recommended by WHO as an approach to tackling this problem [27]. Conventional antisera raised in immunized animals remains the mainstay for snakebite therapy, but they are heterogeneous formulations of variable quality [28]. Antibody phage display technology offers a rapid, low-cost route to the generation of effective therapeutics that are suited to local requirements. 

Currently, antivenoms are manufactured from animal plasma, generally from horses and sheep, and administered as monovalent or polyvalent immunoglobulins, Fab or F(ab)2 fragments, but a range of adverse reactions are observed [29, 30]. Phage display technology and the production of recombinant antibodies in bacteria, yeast, insect, and mammalian expression systems have the potential to overcome these contraindications [13] and studies of recombinant antibodies against venoms from snakes [31, 32] and scorpions [33, 34] show the feasibility of this approach. While phage display is usually applied to antibody discovery, other non-antibody scaffolds also can be considered for the design of anti-venoms [35].

*Naja oxiana*, the Caspian cobra, is an elapid snake of medical importance that can be found in Iran, Afghanistan, and Pakistan. The fractions of *Naja oxiana* venom isolated for this study contained proteins of less than 20 kDa. The major toxic components are from three finger toxins and phospholipase A_2_ enzymes. Research has shown that antibodies raised against proteins in these fractions are able to neutralize the crude venom [36]. Our data and other preliminary findings indicate that fraction F3 has a somewhat lower lethal potency than F4, which is due to high PLA_2_ enzyme activity. In contrast, the higher toxicity of F4 derives from neurotoxin activity rather than PLA_2_. 

The capacity of a monoclonal antibody to neutralize a neurotoxin from snake venom was established some time ago [37]. More recent work has focused on antibodies from recombinant sources in a study with the neurotoxin from *Naja kaouthia* venom [38] and other studies have focused on the α-cobrotoxin of this species using phage libraries and a penta-body configuration to develop an antivenom [39]. PLA_2_ in *N. kaouthia* venom is also an important component which has been targeted in phage display, resulting in effective neutralizing agents [40]. 

## Conclusion

The present work reports the isolation of recombinant antibodies of a human scFv library directed against toxic fractions from *Naja oxiana* venom. The studied clones appear able to neutralize the crude venom at modest levels as evaluated through *in vivo* challenge. The properties of these scFvs may be improved through *in vitro* mutagenesis to enhance their neutralizing potential.

### Abbreviations

CDRs: complementarity-determining regions; IMAC: immobilized nickel affinity chromatography; PLA_2_: phospholipase A_2._
